# Using Accelerated Molecular Dynamics Simulation to elucidate the effects of the T198F mutation on the molecular flexibility of the West Nile virus envelope protein

**DOI:** 10.1038/s41598-020-66344-8

**Published:** 2020-06-15

**Authors:** Renan Patrick da Penha Valente, Rafael Conceição de Souza, Gabriela de Medeiros Muniz, João Elias Vidueira Ferreira, Ricardo Morais de Miranda, Anderson Henrique Lima e Lima, João Lídio da Silva Gonçalves Vianez Junior

**Affiliations:** 1 Center for Technological Innovation, Evandro Chagas Institute, Ministry of Health, Ananindeua, PA 67030-000 Brazil; 2 Federal Institute of Education, Science and Technology of Pará, Tucuruí, PA 68455695 Brazil; 3 Laboratório de Planejamento e Desenvolvimento de Fármacos, Instituto de Ciências Exatas e Naturais, Universidade Federal do Pará, 66075-110 Belém, PA Brasil

**Keywords:** Molecular conformation, West nile virus, Virus structures, Immune evasion

## Abstract

The envelope (E) protein is an important target for antibodies in flavivirus. Literature reports that the mutation T198F, located at the domain I-II hinge of the E protein, regulates viral breathing and increases the accessibility of a distal cryptic epitope located on the fusion loop, having a direct impact in the neutralization of West Nile virus (WNV). Our study aimed to describe, using accelerated molecular dynamics simulations, the effects of the T198F mutation in the flexibility of the E protein of WNV and to elucidate the mechanism that regulates epitope accessibility. The simulation results revealed that the mutation favors the formation of alternative hydrogen bonds, hampering the bending movement between domains I and II. We hypothesized that this is the mechanism by which the T198F mutation, located at the middle of the protein, locks the distal cryptc epitope near a single preferred conformation, rendering it more prone to recognition by antibodies.

## Introduction

West Nile virus (WNV) is a neurotropic human pathogen commonly found in the African region, as well as in Europe, Middle East, Americas and West Asia. The most frequent way of transmission to humans occurs by the bite of infected mosquitoes. Birds are considered its main reservoir, while humans and other mammals are considered incidental hosts^1,2^. The virus was discovered in a febrile adult woman in the West Nile district of Uganda in 1937^3^. Since its discovery, many cases have been reported and today the virus represents a real threat to humans, equines and wildlife, mainly because no vaccine is available^1,4,5^.

WNV is a member of the *flaviviridae* family, genus flavivirus. Like all flaviviruses, it has a single stranded positive sense RNA, encoding structural - Capsid (C), pre-membrane (prM) and Envelope (E) - and non-structural - NS1, NS2A, NS2B, NS3, NS4A, NS4B, NS5 - proteins^6,7^. The E protein is responsible for virus entry^8,9^ and presents three structural domains (DI, DII and DIII)^11^. There are 180 copies of the envelope protein arranged as antiparallel dimmers which are distributed on the surface of mature virions, so it is a major target for neutralizing antibodies^8^.

Neutralization studies suggest that the T198F mutation alone can regulate WNV conformation dynamics (“viral breathing”), having a significant impact in the exposure of a cryptic epitope, modulating antibody recognition potency. This epitope is targeted by the monoclonal antibody E60 (and henceforth referred as epitope E60) and is located on the distal fusion loop (FL) of the envelope protein^10^.

Although Goo *et al*. hypothesized that the T198F mutation increased the accessibility of E60 due to changes in the conformational dynamics of the E protein, they could not infer the structural basis of this phenomena from their data^10^. The elucidation of the underlying mechanisms leading to alterations in molecular flexibility and antibody recognition can be achieved using molecular modeling methods, with techniques such as classical Molecular Dynamics (cMD) and accelerated Molecular Dynamics (aMD) simulations^11–13^. Regarding the conformations of protein macrostructures, aMD simulation scales have greater efficiency when compared to MD simulation, for example, an aMD simulation of 500 ns can be equivalent to about 1 million nanoseconds on the MD scales, depending on the system^12^.

Our study aimed to investigate, using aMD simulations, the molecular flexibility of the envelope protein of WNV. Two systems, wild (T198) and mutant (T198F) were simulated in order to gain insight into the possible mechanisms that regulate the exposure of the cryptic epitope E60.

## Results

### Estimated parameters for aMD simulation

Classical molecular dynamic (cMD) simulation, one of the first steps necessary to simulate aMD, must give information about total potential energy (EPTOT) and dihedral angle energy (DIHED) since the average of these parameters is used as input in an aMD. In this respect, the variables EPTOT and DIHED remained stable during 10 ns of cMD simulation considering both wild and mutant systems of the envelope protein from WNV (See Fig. S1). The averages for EPTOT were -576560.0293 kcal/mol and -575954.3888 kcal/mol for T198 and T198F systems, respectively. The averages DIHED were 4863.0261 kcal/mol and 4902.0074 kcal/mol, respectively. Values for the calculated parameters used as input in aMD simulation and also the variables to calculate them can be found in Table S1.

### Root Mean Square Deviation (RMSD) and Root Mean Square Fluctuation (RMSF) analysis indicated that the T198F mutation hampers the flexibility of the E protein, mainly in structural domain II

**Figure 1:**
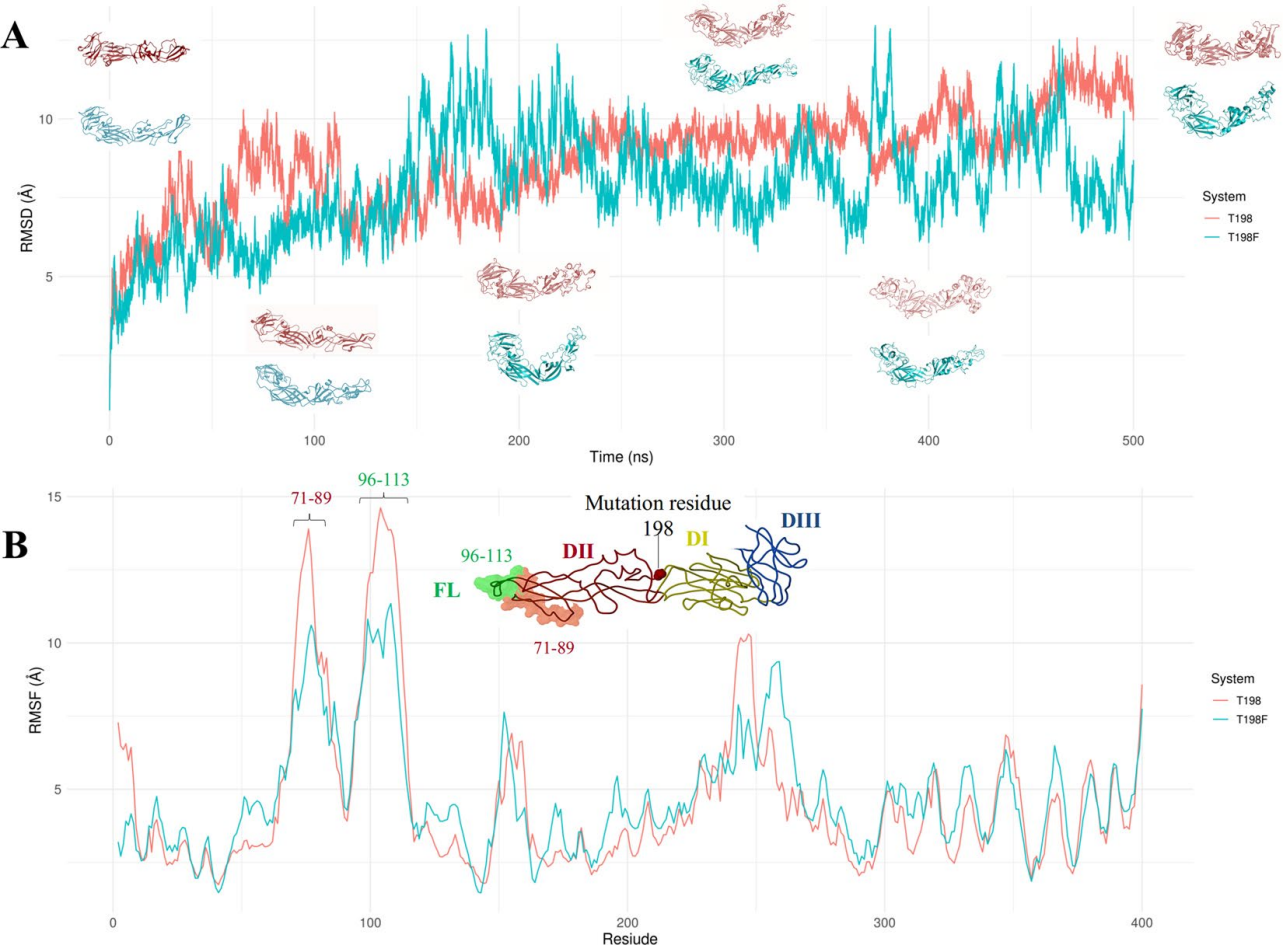
RMSD (**A**) and RMSF (**B**) during 500 ns of aMD simulation. In (**A**), changes in conformations are illustrated for different times (0, 100, 200, 300, 400 and 500 ns), showing that the T198F system presents lower RMSD values, suggesting limited changes in conformation. In (**B**), the residues with more fluctuations are displayed (71–89 and 96–113). It is also shown the illustration of the envelope protein, depicting DI (yellow), DII (red), DIII (blue) and the fusion loop (FL, green). The green shaded region comprises the residues 96–113 (FL) while the red shaded region comprises the residues 71–89.

During 500 ns of aMD simulation, the RMSD analysis was used to measure conformational changes in the E protein, using as reference the structure obtained from the last frame of the 10 ns of cMD. On the other hand, RMSF was employed to quantify the fluctuation in each residue of both systems, allowing to identify the most flexible regions. The RMSD analysis shows that there are more structural deviations in the T198 system, suggesting reduced flexibility in the mutant system (Fig. 1). RMSF analysis allowed us to identify that the increased flexibility in the T198 system was mainly associated with two regions of the structural domain II (DII). One of the regions involves the residues 71–89, whose average RMSF values were about 15 Å for wild and 10 Å for the T198F system, respectively. The other region includes residues 96–113, whose average RMSF values were similar to the T198, with a small increase. These regions in DII include the fusion loop (FL) and E60.

### Dynamical cross-correlation analysis shows that the T198F mutation decreases correlated movements in the E protein of WNV

**Figure 2:**
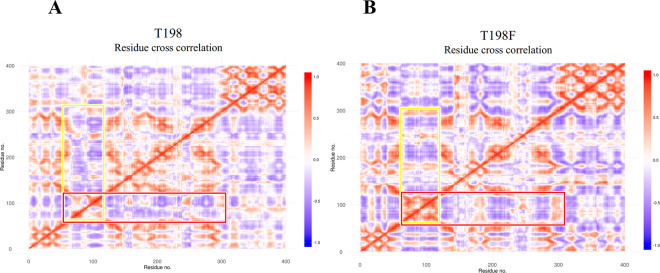
Cross-correlation map (prepared using R software^14^) for aMD simulation: (**A**) T198 system and (**B**) T198F system, region of colored rectangles include residues 71–89, 96–113 and 130–280.

A dynamic cross-correlation matrix (DCCM) was generated to identify correlated or anti-correlated movements^12^ in both systems. From the resulting matrix, we could infer that residues 71–89 and 96–113 shows a considerable correlated movement with residues 130–280 (colored rectangles, Fig. 2A). In the mutated system, was observed a loss of those correlated movements in some regions, and significant increases of anti-correlated movements in the other system (Fig. 2B). The region 130–280 corresponds to residues that are part of DI-DII, while regions 93–113 and 71–89 corresponds to the fusion loop (FL) and to a loop adjacent to FL, respectively. In general, after mutation, the protein revealed an increase in correlated and anti-correlated movements, this should be related to exposure of the epitope.

### PCA analysis indicated that the T198F mutation changes conformational minimum states

**Figure 3:**
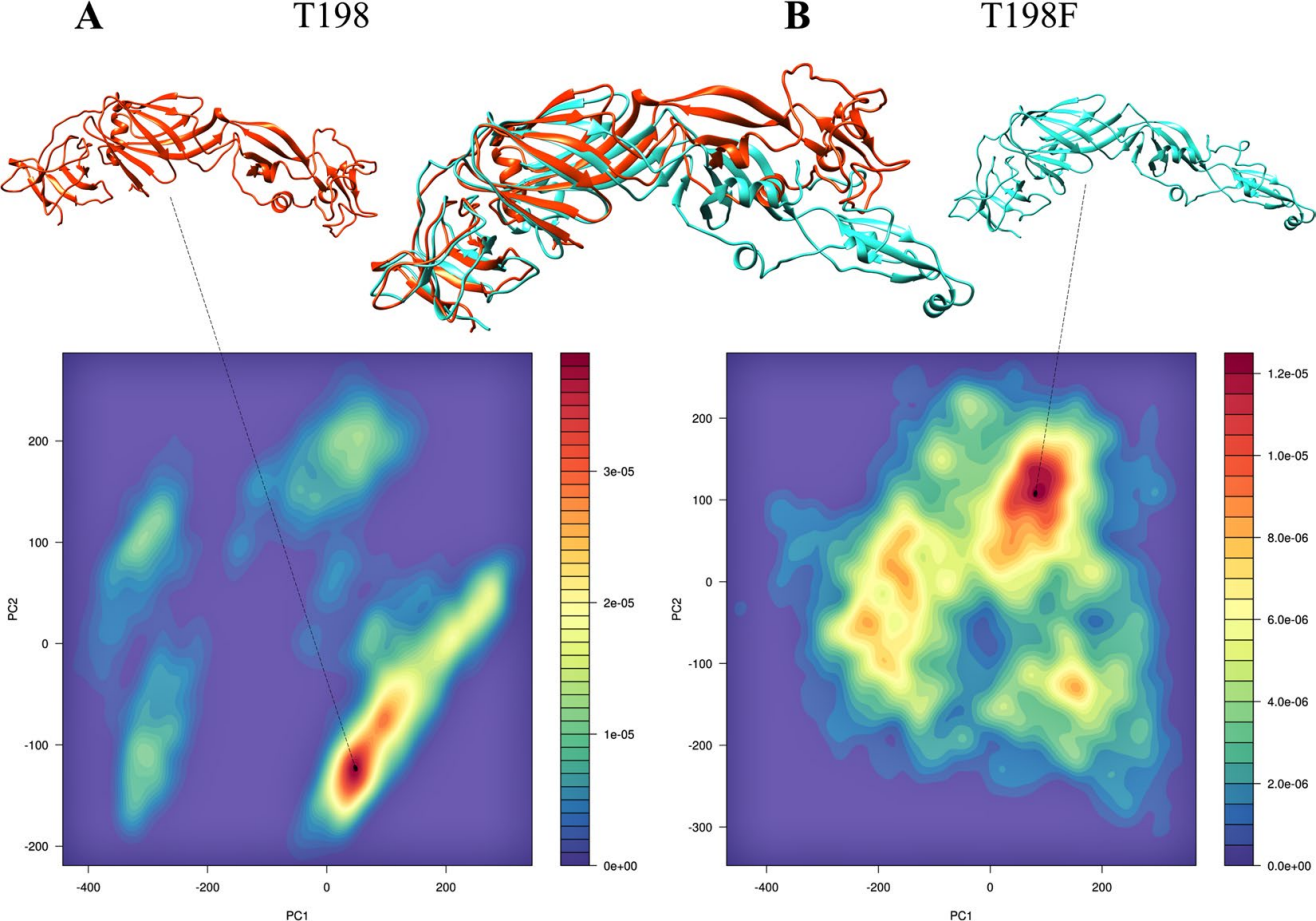
Histogram for T198 (**A**) and T198F (**B**) systems. In the middle is the alignment of the minimal ensemble conformations of the systems A and B. Protein images prepared using UCSF Chimera^15^ and PC1 vs PC2 using R software^14^.

Based on principal components and free energy (Fig. S2, support information), histograms were projected to identify the preferable conformations of the two systems. Differences in the conformations observed in each basin depict a bending movement between DI and DII, which is particular of the E protein. This data corroborates the RMSF and cross-correlations analysis, suggesting a restriction in the flexibility of the T198F system, that quickly reaches a stable ensemble of conformations. Figure 3 presents minimal conformations for the systems T198 and T198F, the alignment between the average ensemble of conformations, clearly demonstrates that the mutation exposes FL, as well as the Epitope E60.

### Hydrogen bond investigation explains the ensemble of preferential conformations

**Figure 4:**
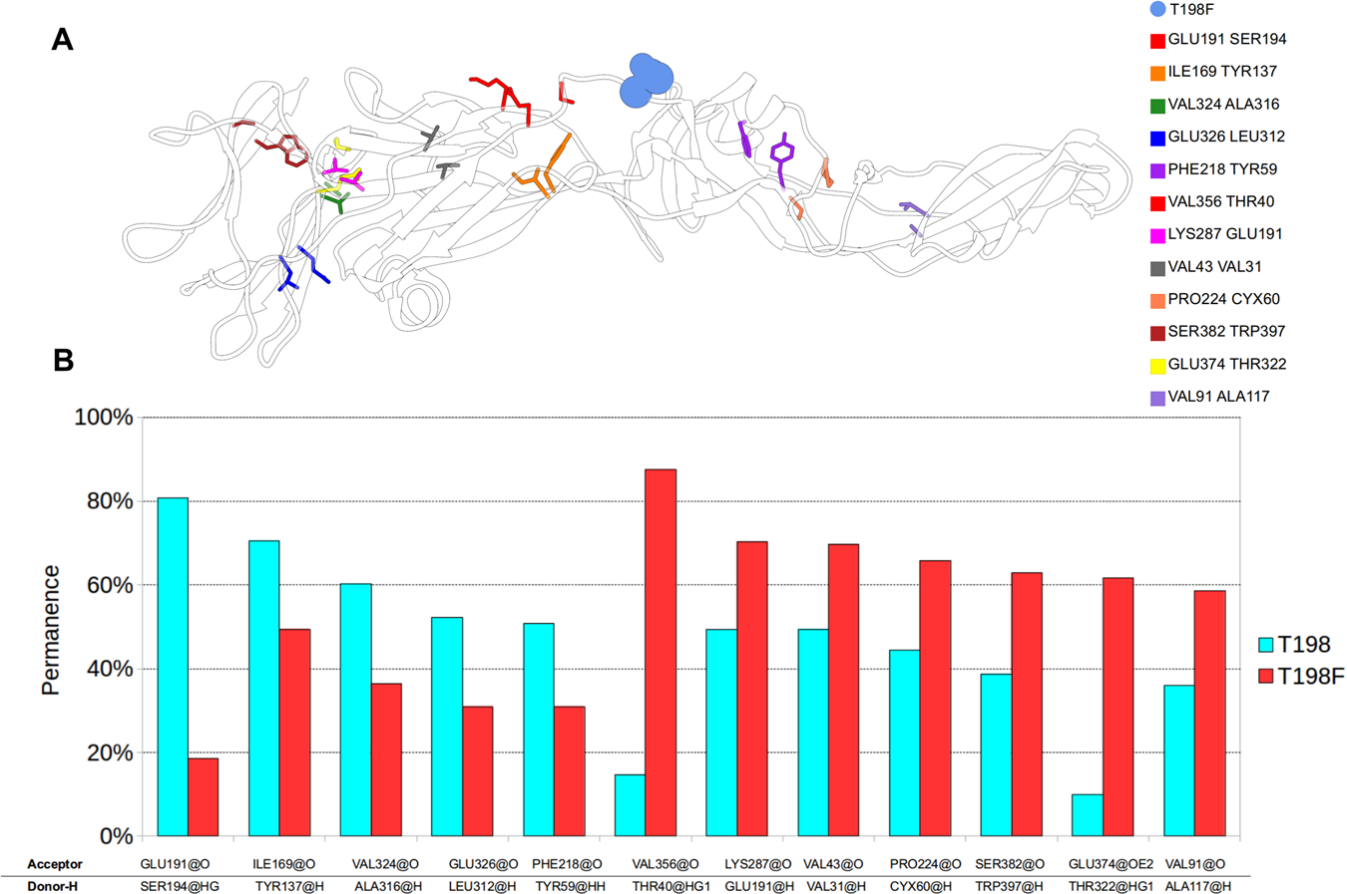
(**A**) Bonds that reduces the flexibility of the E protein of WNV in DI, DII and DIII (prepared in UCSF Chimera^15^), and **(B)** permanence in 500 ns of simulation.

*In silico* investigation of hydrogen bonds were identified some key interactions to explain the minimum ensembles of conformations (Fig. 4). We considered as stable hydrogen bonding interactions those that were maintained for>50% of the total simulation time. The hydrogen bond interaction between the residues V356-T40 was the most effective in the mutant system, being established > 80% of the simulation. In general, the wild-type system presented more interactions than T198F: 2233 and 2077, respectively (difference = 156). Nevertheless, they were not so effective, since a part of them were not considered stable (>50% of the simulation time). The higher stability of alternative hydrogen bonds after mutation (Fig. 4) explains the loss of movements of the T198F system, leading to the observed changes in the conformational minimum states.

## Discussion

Our simulations revealed that the envelope protein in its native state (T198 system) can explore the minimums of the conformational space with more difficulty in comparison to the mutated (T198F) system, which is less stable (greater molecular flexibility), agreeing with the hypothesis of altered flexibility supported by Goo *et al*.^10^. The changes in the flexibility induced by the T198F mutation were accentuated near the cryptic epitope E60, as was already suggested by them. Besides confirming what was suggested, the present computational simulation predicted that the increased exposure of E60 is due to the reduced flexibility in the T198F system. Even though the T198F mutation is located in the middle of the E protein and far from E60, it allowed the strengthening of particular hydrogen bonds (Fig. 4). This strengthening was able to restrict a bending movement that occurs between DI and DII. In this way, we propose that this is the mechanism by which the T198F mutation exposure the epitope E60 in a minimum ensemble of conformations, rendering this region more susceptible to antibody binding. To summarize, this study demonstrates how the alteration of a single residue in the hinge region of the E protein can influence the dynamics of a distal cryptic epitope, shedding light in the observed differences in neutralization efficiencies and have implications in future attempts of rational planning of vaccines and design antiviral compounds.

## Materials and Methods

### System settings

The envelope protein of WNV used was retrieved from the protein data bank (PDB, www.rcsb.org)^16^ and identified by code 2HG0^11^. Firstly, the amino acid protonation states were settled using H++ (http://biophysics.cs.vt.edu) webserver^17^. The force field employed to describe the protein was the ff99SB^18^ using tLeap module of AMBER 16^19^. The protein was solvated with TIP3P water molecules^20^ and counter-ions were added in order to maintain the electroneutrality of the systems.

### Classical and accelerated molecular dynamics simulations

Energy minimization, equilibration and system heating were carried out with AMBER 16 package as described previously by our group^21^. Firstly, a five-step minimization protocol was applied. During stages 1–3, all heavy atoms were restrained using a harmonic constant of 1000 kcal mol^−1^ Å^2^ while applying 5000 and 10000 steps of steepest-descent (SD) and conjugate-gradients (CG), respectively. In step 4, 30000 steps of SD and 35000 steps of CG were used, while maintaining only solute heavy atoms restrained. During step 5, 5000 steps of SD and 50000 steps of CG were applied with all atoms free to move. Afterward, a preliminary MD (2 ns) with all heavy atoms positions restrained (25 kcal mol^−1^ Å) was performed to heat each system to 310 K in NVT employing Langevin dynamics as thermostat (collision frequency of 2 ps). SHAKE algorithm was employed to constraints all bonds involving hydrogen atoms and the Particle Mesh Ewald method^22^ was employed to calculate the electrostatic interactions of long distances with a cutoff radius of 10 Å.

The average dihedral and total potential energies were computed during a 10 ns classical molecular dynamics simulation and taken as reference for the aMD simulations. The aMD modifies the original dihedral potential (V(r)) by adding an increment, Δ*V*(*r*) when V(r) is below a defined energy level E^19,23^, as in Eq. 1:

1 $$\Delta V(r)=\{0,\begin{array}{c}\frac{{(E-V(r))}^{2}}{\propto +(E-V(r))}\end{array}\cdot \frac{V(r)\ge E}{V(r) < E}$$

Where α modules the depth and roughness of the energy valleys in the modified potential.

The parameters E and ∝ were the calculated average energy for the dihedral and total potential energy obtained at the end of 10 ns of simulation of classic MD^19,23^.

### Analysis of the simulations

To compute the root mean square deviation (RMSD) and the root mean square fluctuation (RMSF) for both trajectories, the CPPTRAJ module of AmberTools 16 was used, considering only alpha carbon atoms^24^ according to equations described by Romanowska and coworkers in 2008 (Eq. 2 and Eq. 3):

2 $${\rm{RMSD}}({t}_{1},{t}_{2})=\sqrt{\frac{1}{M}{\sum }_{{\rm{i}}=1}^{{\rm{N}}}{{\rm{m}}}_{{\rm{i}}}||\overrightarrow{{r}_{i}}({t}_{1})-\overrightarrow{{r}_{i}}({t}_{2}){||}^{2}}$$

3 $${\rm{RMSF}}(i)=\sqrt{\frac{1}{T}{\sum }_{t=1}^{T}||\overrightarrow{{r}_{i}}(t)-\overrightarrow{{r}_{i}}|{|}^{2}}$$

#### Principal component analysis (PCA) and free energy landscape (FEL)

PCA is a well-known technique used to extract large-scale concerted movements in biomolecules. Briefly, a covariance matrix C_ij_ of the fluctuation of the alpha carbons is calculated and diagonalized, using the molecular dynamics trajectory as an input. The elements of the covariance matrix are:

4 $${C}_{ij}=({x}_{i}-{x}_{j})(i,j=1,2,3,\ldots ,3N)$$

Where *i* and *j* denote all pairs of the 3 N cartesian coordinates. x_i_ and x_j_ are instantaneous values of the i-th and j-th alfa carbon atom, respectively. N is the number of atoms considered and x_i_ and x_j_ represent the average value in all configurations obtained in the aMD^25^. Dynamical cross-correlation matrices (DCCM), principal component and FEL analysis (PCA) were computed using the Bio3D package^26^ in R software^14^.

## Supplementary information

Supplemental information.
