# Supplementary material for: Using Accelerated Molecular Dynamics Simulation to elucidate the effects of the T198F mutation on the molecular flexibility of the West Nile virus envelope protein
Source: Sci Rep. 2020 Jun 15;10:9625. doi: 10.1038/s41598-020-66344-8 (PMC7296010; doi:10.1038/s41598-020-66344-8)
Supplement: Supplementary file 1 — Supplemental information. [file 41598_2020_66344_MOESM1_ESM.docx]

**Using Accelerated Molecular Dynamics Simulation to elucidate the likely effects of a T198F mutation on the molecular flexibility of the West Nile virus envelope protein**

Renan Patrick da Penha Valente**^ac^**, Rafael Conceição de Souza**^a^**, Gabriela de Medeiros Muniz**^a^**, João Elias Vidueira Ferreira**^b^**, Ricardo Morais de Miranda**^b^**, Anderson Henrique Lima e Lima**^c,^** and João Lídio da Silva Gonçalves Vianez Junior**^a*^**

**^a^** Center for Technological Innovation. Evandro Chagas Institute. Ministry of Health, Ananindeua, PA 67030-000, Brazil

**^b^** Federal Institute of Education, Science and Technology of Pará, Tucuruí, PA 68455695, Brazil

**^c^** Laboratório de Planejamento e Desenvolvimento de Fármacos, Instituto de Ciências Exatas e Naturais, Universidade Federal do Pará, 66075-110, Belém, PA, Brasil.

**Supporting information**

**Table S1**. Variables and parameters used in the aMD simulation for all systems. Ab is the abbreviation for antibody.

| **Systems** | | **T198** | **T198F** | **Wild-ab** | **T198F-ab** |
| --- | --- | --- | --- | --- | --- |
| **Variables** | Residues | 400 | 400 | 819 | 819 |
|  | Total Atoms | 61361 | 61277 | 257299 | 257293 |
|  | Water | 60959 | 60874 | 81659 | 81655 |
|  | Ions | 2Na^+^ | 3Na^+^ | 2Cl^-^ | 2Cl^-^ |
|  | EPtot (kcal/mol) | -576560.0293 | -575954.3888 | -1356103.5046 | -772998,362 |
|  | DIHED (kcal/mol) | 4863.0261 | 4902.0074 | 10008.2200 | 10083,1295 |
| **Calculated Parameters** | Ethreshd | 6502.0074 | 6481.9429 | 13284.22 | 13364.584 |
|  | Alphad | 320 | 320 | 655.2 | 655.2 |
|  | Ethreshp | -546337.1493 | -545771.3488 | −1314935,6646 | −731850,8025 |
|  | Alphap | 30222.88 | 30183.04 | 41167,84 | 41166,88 |

**Figure S1.** EPTOT and DIHED during 10ns of cMD for the isolated protein. Potential energies maintained stable during 10 ns, giving credibility to the simulation.


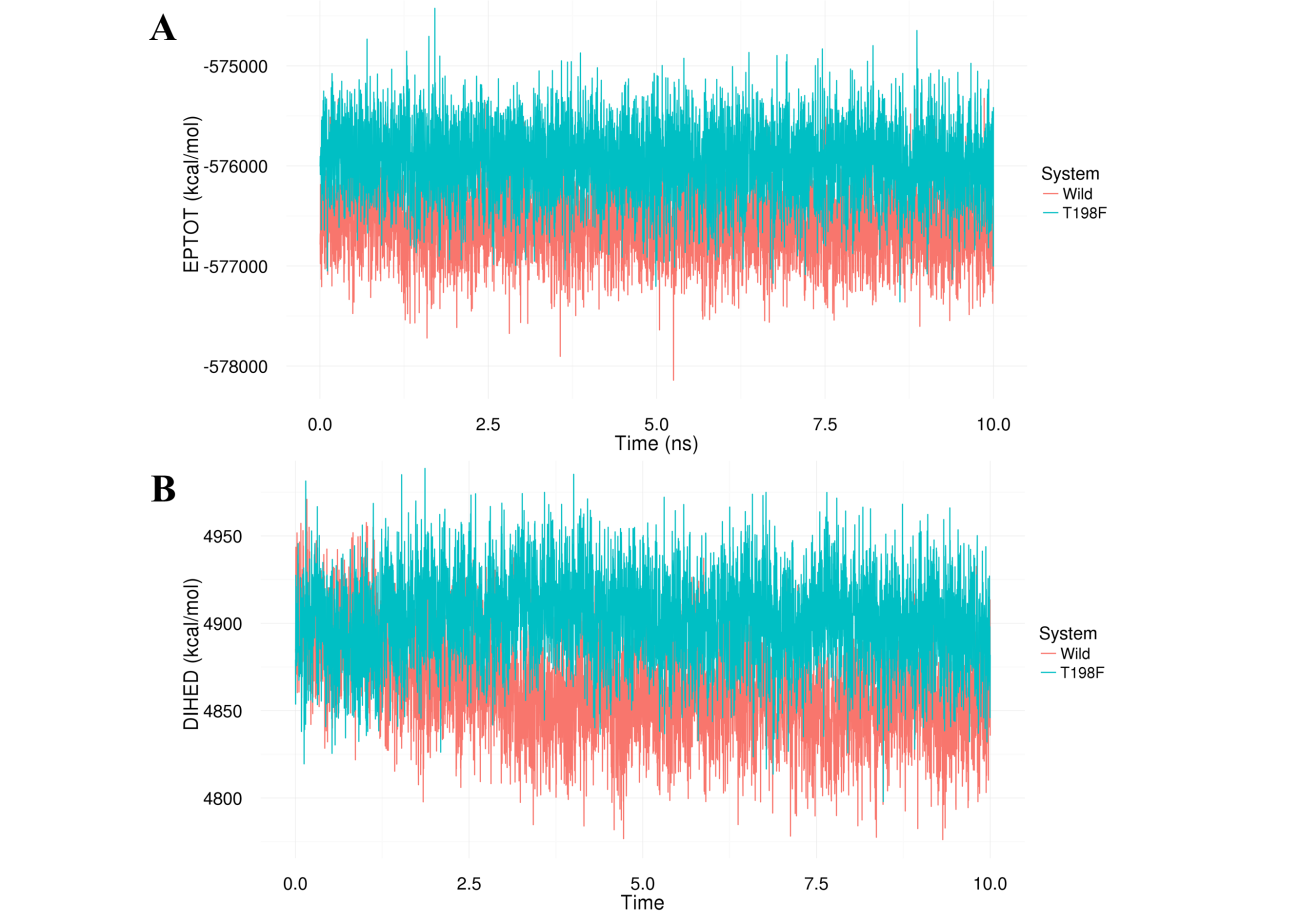


Figure S2: PCA data

| 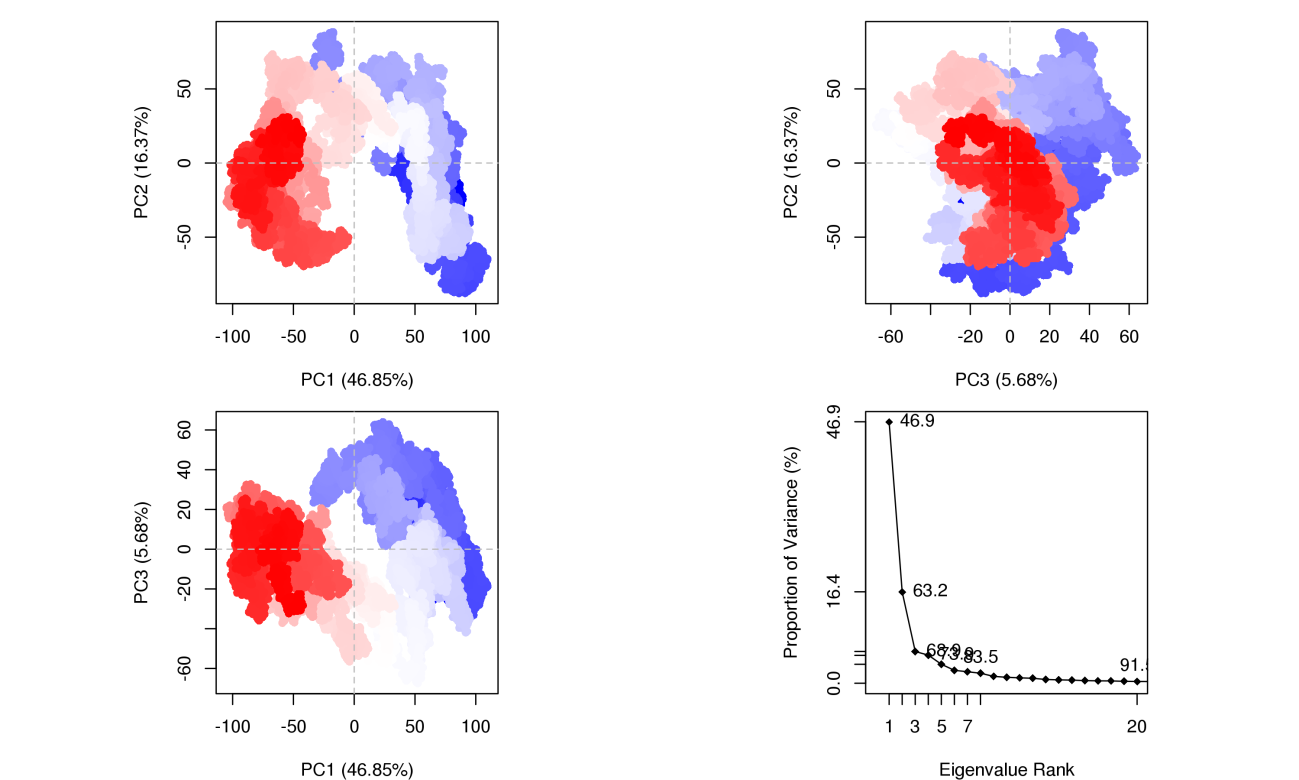   1. T198 |
| --- |
| 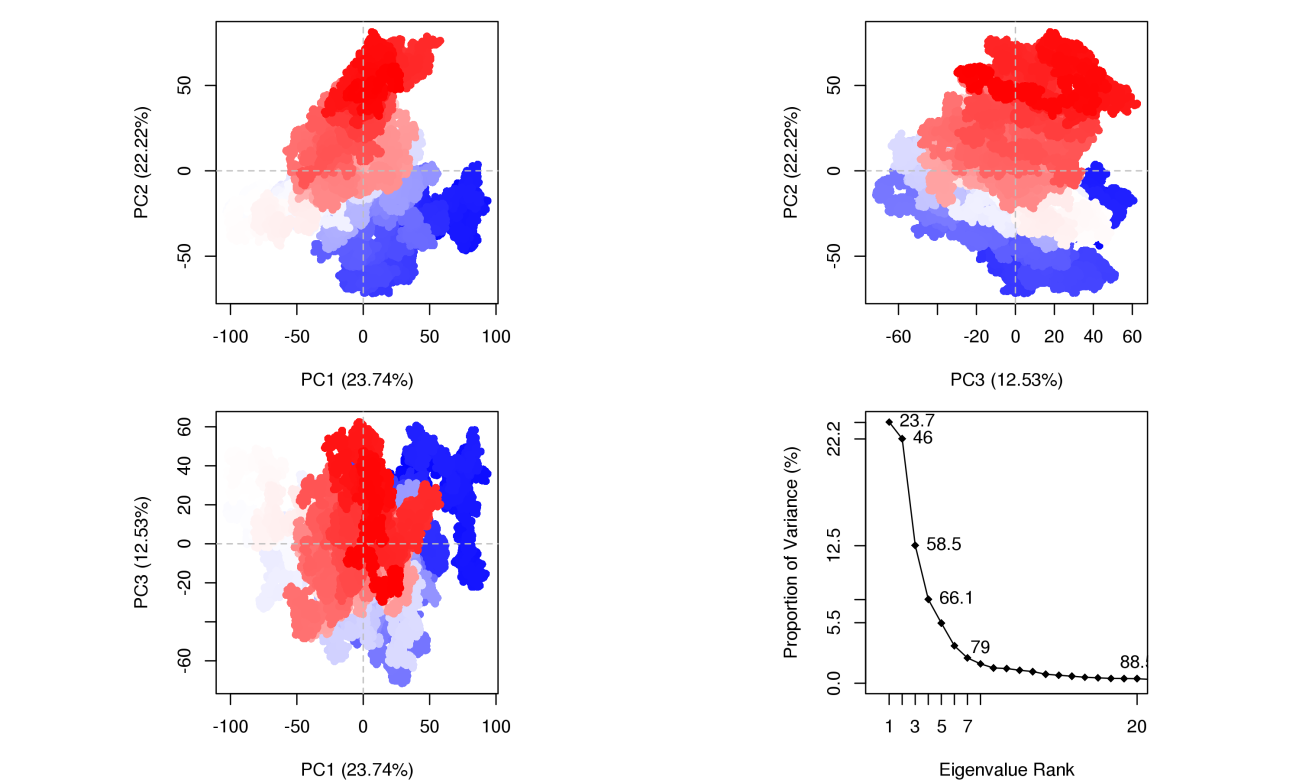   1. T198F |

**Table S2.** Main hydrogen bonds (reported in Figure 4).

| **System** | **Acceptor** | **Donor-H** | **Donor** | **AVG distance (Å)** | **Avg Ang** | **Permanence** |
| --- | --- | --- | --- | --- | --- | --- |
| **T198** | GLU191@O | SER194@HG | SER194@OG | 2.7492 | 160.4701 | 0.8074 |
|  | ILE169@O | TYR137@H | TYR137@N | 2.8535 | 160.2705 | 0.7059 |
|  | VAL324@O | ALA316@H | ALA316@N | 2.8337 | 155.7630 | 0.6032 |
|  | GLU326@O | LEU312@H | LEU312@N | 2.8583 | 154.7384 | 0.5223 |
|  | PHE218@O | TYR59@HH | TYR59@OH | 2.7254 | 161.1582 | 0.5077 |
|  | VAL356@O | THR40@HG1 | THR40@OG1 | 2.7588 | 158.9934 | 0.1466 |
|  | LYS287@O | GLU191@H | GLU191@N | 2.8817 | 160.8704 | 0.4936 |
|  | VAL43@O | VAL31@H | VAL31@N | 2.8780 | 160.5082 | 0.4946 |
|  | PRO224@O | CYX60@H | CYX60@N | 2.8492 | 160.5920 | 0.4440 |
|  | SER382@O | TRP397@H | TRP397@N | 2.8682 | 159.5987 | 0.3874 |
|  | GLU374@OE2 | THR322@HG1 | THR322@OG1 | 2.7058 | 163.4132 | 0.0997 |
|  | VAL91@O | ALA117@H | ALA117@N | 2.8656 | 158.8811 | 0.3595 |
| **T198F** | GLU191@O | SER194@HG | SER194@OG | 2.6830 | 163.0848 | 0.1853 |
|  | ILE169@O | TYR137@H | TYR137@N | 2.8629 | 160.5158 | 0.4945 |
|  | VAL324@O | ALA316@H | ALA316@N | 2.8717 | 159.0720 | 0.3645 |
|  | GLU326@O | LEU312@H | LEU312@N | 2.8700 | 157.0741 | 0.3098 |
|  | PHE218@O | TYR59@HH | TYR59@OH | 2.8700 | 157.0741 | 0.3098 |
|  | VAL356@O | THR40@HG1 | THR40@OG1 | 2.7389 | 160.9704 | 0.8763 |
|  | LYS287@O | GLU191@H | GLU191@N | 2.8498 | 160.8035 | 0.7035 |
|  | VAL43@O | VAL31@H | VAL31@N | 2.8570 | 160.2790 | 0.6973 |
|  | PRO224@O | CYX60@H | CYX60@N | 2.8437 | 160.5464 | 0.6572 |
|  | SER382@O | TRP397@H | TRP397@N | 2.8469 | 157.5198 | 0.6284 |
|  | GLU374@OE2 | THR322@HG1 | THR322@OG1 | 2.6782 | 164.9523 | 0.6160 |
|  | VAL91@O | ALA117@H | ALA117@N | 2.8643 | 157.7543 | 0.5856 |

**S3 Table**. T198 – All H-bonds (Permanence > 0.50)

| **Acceptor** | **Donor-H** | **Donor** | **AVG distance (Å)** | **Avg Ang** | **Permanence** |
| --- | --- | --- | --- | --- | --- |
| GLU191@O | SER194@HG | SER194@OG | 2.7492 | 160.4701 | 0.8074 |
| GLU138@O | MET46@H | MET46@N | 2.8308 | 157.9921 | 0.7890 |
| HID395@O | ILE384@H | ILE384@N | 2.8464 | 161.1677 | 0.7748 |
| VAL203@O | ARG128@H | ARG128@N | 2.8413 | 160.0421 | 0.7657 |
| LYS44@O | ALA140@H | ALA140@N | 2.8499 | 160.7585 | 0.7636 |
| ILE41@O | ILE33@H | ILE33@N | 2.8416 | 158.3050 | 0.7446 |
| PHE211@O | MET204@H | MET204@N | 2.8565 | 161.2186 | 0.7357 |
| ILE270@O | LEU212@H | LEU212@N | 2.8481 | 161.1986 | 0.7272 |
| ALA200@O | ARG215@H | ARG215@N | 2.8381 | 159.0266 | 0.7262 |
| GLU49@O | LYS136@H | LYS136@N | 2.8436 | 160.4274 | 0.7252 |
| PHE142@O | ASP42@H | ASP42@N | 2.8565 | 161.5896 | 0.7245 |
| LYS310@O | GLN328@H | GLN328@N | 2.8535 | 160.7580 | 0.7227 |
| LEU286@O | LEU25@H | LEU25@N | 2.8561 | 161.3218 | 0.7171 |
| MET204@O | PHE211@H | PHE211@N | 2.8620 | 161.6274 | 0.7065 |
| ILE169@O | TYR137@H | TYR137@N | 2.8535 | 160.2705 | 0.7059 |
| VAL213@O | TYR202@H | TYR202@N | 2.8563 | 160.2425 | 0.7043 |
| THR187@O | LYS291@H | LYS291@N | 2.8587 | 161.0700 | 0.7035 |
| ASP189@O | ARG289@H | ARG289@N | 2.8509 | 158.3990 | 0.6999 |
| ARG354@O | GLU376@H | GLU376@N | 2.8581 | 162.2650 | 0.6972 |
| LEU375@O | VAL323@H | VAL323@N | 2.8512 | 159.6737 | 0.6872 |
| ALA54@O | THR129@H | THR129@N | 2.8470 | 158.3366 | 0.6833 |
| CYX336@O | VAL362@H | VAL362@N | 2.8580 | 160.4579 | 0.6726 |
| TYR202@O | VAL213@H | VAL213@N | 2.8668 | 162.5204 | 0.6711 |
| TYR383@O | VAL343@H | VAL343@N | 2.8619 | 160.8888 | 0.6691 |
| SER341@O | VAL385@H | VAL385@N | 2.8678 | 162.2264 | 0.6673 |
| ASP10@O | THR32@H | THR32@N | 2.8565 | 161.0356 | 0.6618 |
| VAL21@O | VAL290@H | VAL290@N | 2.8645 | 160.2096 | 0.6617 |
| LEU327@O | VAL371@H | VAL371@N | 2.8592 | 159.5503 | 0.6583 |
| ILE373@O | LEU325@H | LEU325@N | 2.8659 | 161.5480 | 0.6572 |
| VAL186@O | LEU178@H | LEU178@N | 2.8563 | 159.5214 | 0.6534 |
| VAL139@O | PHE167@H | PHE167@N | 2.8641 | 160.5433 | 0.6518 |
| GLY184@O | LEU180@H | LEU180@N | 2.8430 | 157.9097 | 0.6518 |
| TYR176@O | VAL188@H | VAL188@N | 2.8635 | 161.8296 | 0.6493 |
| VAL279@O | ALA50@H | ALA50@N | 2.8611 | 159.1359 | 0.6416 |
| GLY303@O | LYS337@H | LYS337@N | 2.8469 | 160.4849 | 0.6393 |
| ALA125@O | TYR59@H | TYR59@N | 2.8439 | 157.7697 | 0.6336 |
| PRO360@O | VAL338@H | VAL338@N | 2.8641 | 161.7423 | 0.6311 |
| ARG289@O | ASP189@H | ASP189@N | 2.8713 | 160.9218 | 0.6303 |
| ALA369@O | TYR329@H | TYR329@N | 2.8580 | 160.7241 | 0.6295 |
| ASN47@O | GLU138@H | GLU138@N | 2.8573 | 158.8818 | 0.6254 |
| LEU12@O | MET34@H | MET34@N | 2.8528 | 158.5667 | 0.6223 |
| TYR137@O | ILE169@H | ILE169@N | 2.8628 | 160.1294 | 0.6153 |
| THR32@O | LEU12@H | LEU12@N | 2.8641 | 159.0145 | 0.6091 |
| LEU325@O | ILE373@H | ILE373@N | 2.8728 | 161.5658 | 0.6076 |
| LYS136@O | GLU49@H | GLU49@N | 2.8643 | 160.4746 | 0.6071 |
| VAL324@O | ALA316@H | ALA316@N | 2.8337 | 155.7630 | 0.6032 |
| TYR59@O | ALA125@H | ALA125@N | 2.8528 | 160.2166 | 0.5987 |
| LYS209@O | VAL206@H | VAL206@N | 2.8564 | 159.8600 | 0.5975 |
| CYX288@O | LEU23@H | LEU23@N | 2.8664 | 159.3084 | 0.5952 |
| VAL343@O | TYR383@H | TYR383@N | 2.8609 | 160.2811 | 0.5942 |
| VAL31@O | VAL43@H | VAL43@N | 2.8748 | 160.2576 | 0.5871 |
| VAL290@O | VAL21@H | VAL21@N | 2.8666 | 159.5520 | 0.5864 |
| ILE393@O | VAL386@H | VAL386@N | 2.8770 | 162.0900 | 0.5816 |
| GLN328@O | LYS310@H | LYS310@N | 2.8670 | 158.9190 | 0.5742 |
| ILE126@O | THR205@H | THR205@N | 2.8715 | 160.0012 | 0.5644 |
| VAL371@O | LEU327@H | LEU327@N | 2.8769 | 161.3418 | 0.5632 |
| THR210@OG1 | THR205@HG1 | THR205@OG1 | 2.8125 | 163.0864 | 0.5608 |
| VAL323@O | LEU375@H | LEU375@N | 2.8793 | 161.5692 | 0.5607 |
| GLY165@O | ILE141@H | ILE141@N | 2.8740 | 161.1629 | 0.5545 |
| PHE167@O | VAL139@H | VAL139@N | 2.8768 | 161.5197 | 0.5509 |
| GLU273@O | LYS280@H | LYS280@N | 2.8635 | 161.5518 | 0.5497 |
| LYS337@O | CYX305@H | CYX305@N | 2.8638 | 161.6871 | 0.5440 |
| THR357@OG1 | GLU374@H | GLU374@N | 2.8786 | 160.7035 | 0.5431 |
| GLU374@O | VAL356@H | VAL356@N | 2.8598 | 153.1296 | 0.5417 |
| ASP42@O | PHE142@H | PHE142@N | 2.8768 | 159.8593 | 0.5414 |
| ARG128@O | VAL203@H | VAL203@N | 2.8740 | 159.5045 | 0.5266 |
| LEU25@O | LEU286@H | LEU286@N | 2.8654 | 159.1026 | 0.5233 |
| GLU326@O | LEU312@H | LEU312@N | 2.8583 | 154.7384 | 0.5223 |
| THR205@O | ILE126@H | ILE126@N | 2.8611 | 157.2564 | 0.5222 |
| ILE384@O | HID395@H | HID395@N | 2.8802 | 157.8770 | 0.5215 |
| PRO339@O | GLY387@H | GLY387@N | 2.8719 | 161.1069 | 0.5191 |
| ARG57@O | GLY127@H | GLY127@N | 2.8443 | 156.4205 | 0.5177 |
| GLY127@O | VAL56@H | VAL56@N | 2.8669 | 158.9968 | 0.5173 |
| VAL386@O | ILE393@H | ILE393@N | 2.8742 | 160.5262 | 0.5155 |
| VAL188@O | TYR176@H | TYR176@N | 2.8635 | 158.5496 | 0.5101 |
| VAL385@O | SER341@H | SER341@N | 2.8804 | 158.5081 | 0.5090 |
| PHE218@O | TYR59@HH | TYR59@OH | 2.7254 | 161.1582 | 0.5077 |

**S4 Table**. T198F – All H-bonds (Permanence > 0.50)

| **Acceptor** | **Donor-H** | **Donor** | **Avg Ang** | **AVG distance (Å)** | **Permanence** |
| --- | --- | --- | --- | --- | --- |
| VAL_356@O | THR_40@HG1 | THR_40@OG1 | 2.7389 | 160.9704 | 0.8763 |
| ARG_354@O | GLU_376@H | GLU_376@N | 2.8388 | 161.9840 | 0.8090 |
| ASP_189@O | ARG_289@H | ARG_289@N | 2.8287 | 158.9199 | 0.8002 |
| LEU_375@O | VAL_323@H | VAL_323@N | 2.8413 | 160.8902 | 0.7971 |
| ILE_41@O | ILE_33@H | ILE_33@N | 2.8349 | 157.9770 | 0.7643 |
| VAL_203@O | ARG_128@H | ARG_128@N | 2.8405 | 159.0411 | 0.7532 |
| CYX_336@O | VAL_362@H | VAL_362@N | 2.8454 | 160.0366 | 0.7469 |
| THR_187@O | LYS_291@H | LYS_291@N | 2.8536 | 161.2869 | 0.7405 |
| LYS_44@O | ALA_140@H | ALA_140@N | 2.8544 | 161.1758 | 0.7359 |
| PHE_211@O | MET_204@H | MET_204@N | 2.8544 | 161.2025 | 0.7344 |
| VAL_213@O | TYR_202@H | TYR_202@N | 2.8446 | 159.2509 | 0.7302 |
| HID_395@O | ILE_384@H | ILE_384@N | 2.8559 | 161.2855 | 0.7295 |
| ILE_270@O | LEU_212@H | LEU_212@N | 2.8509 | 161.1764 | 0.7271 |
| ILE_373@O | LEU_325@H | LEU_325@N | 2.8544 | 161.2192 | 0.7190 |
| GLU_138@O | MET_46@H | MET_46@N | 2.8374 | 156.3946 | 0.7121 |
| ALA_125@O | TYR_59@H | TYR_59@N | 2.8389 | 158.5930 | 0.7039 |
| LYS_287@O | GLU_191@H | GLU_191@N | 2.8498 | 160.8035 | 0.7035 |
| VAL_43@O | VAL_31@H | VAL_31@N | 2.8570 | 160.2790 | 0.6973 |
| LEU_327@O | VAL_371@H | VAL_371@N | 2.8509 | 159.9363 | 0.6964 |
| LEU_286@O | LEU_25@H | LEU_25@N | 2.8565 | 161.1345 | 0.6963 |
| PHE_142@O | ASP_42@H | ASP_42@N | 2.8660 | 162.2491 | 0.6897 |
| TYR_202@O | VAL_213@H | VAL_213@N | 2.8620 | 162.1292 | 0.6864 |
| TYR_59@O | ALA_125@H | ALA_125@N | 2.8463 | 158.9468 | 0.6832 |
| GLY_303@O | LYS_337@H | LYS_337@N | 2.8504 | 161.4532 | 0.6828 |
| MET_204@O | PHE_211@H | PHE_211@N | 2.8670 | 161.6216 | 0.6749 |
| ALA_54@O | THR_129@H | THR_129@N | 2.8510 | 157.8386 | 0.6681 |
| PRO_360@O | VAL_338@H | VAL_338@N | 2.8640 | 162.1413 | 0.6669 |
| TYR_383@O | VAL_343@H | VAL_343@N | 2.8646 | 160.8992 | 0.6657 |
| TYR_176@O | VAL_188@H | VAL_188@N | 2.8635 | 161.7547 | 0.6634 |
| LYS_310@O | GLN_328@H | GLN_328@N | 2.8552 | 159.9482 | 0.6618 |
| ASP_10@O | THR_32@H | THR_32@N | 2.8524 | 160.3450 | 0.6574 |
| PRO_224@O | CYX_60@H | CYX_60@N | 2.8437 | 160.5464 | 0.6572 |
| SER_341@O | VAL_385@H | VAL_385@N | 2.8681 | 162.1880 | 0.6533 |
| VAL_21@O | VAL_290@H | VAL_290@N | 2.8670 | 160.0154 | 0.6426 |
| GLY_184@O | LEU_180@H | LEU_180@N | 2.8484 | 158.7484 | 0.6395 |
| VAL_139@O | PHE_167@H | PHE_167@N | 2.8634 | 160.3392 | 0.6366 |
| GLU_49@O | LYS_136@H | LYS_136@N | 2.8586 | 160.5647 | 0.6362 |
| THR_32@O | LEU_12@H | LEU_12@N | 2.8589 | 159.4158 | 0.6305 |
| VAL_31@O | VAL_43@H | VAL_43@N | 2.8668 | 160.6327 | 0.6303 |
| SER_382@O | TRP_397@H | TRP_397@N | 2.8469 | 157.5198 | 0.6284 |
| VAL_343@O | TYR_383@H | TYR_383@N | 2.8566 | 160.2082 | 0.6272 |
| VAL_186@O | LEU_178@H | LEU_178@N | 2.8623 | 159.9609 | 0.6233 |
| CYX_288@O | LEU_23@H | LEU_23@N | 2.8670 | 160.2280 | 0.6215 |
| THR_40@O | HIE_144@H | HIE_144@N | 2.8762 | 163.2172 | 0.6200 |
| ALA_369@O | TYR_329@H | TYR_329@N | 2.8606 | 159.8532 | 0.6188 |
| LYS_209@O | VAL_206@H | VAL_206@N | 2.8457 | 158.3506 | 0.6177 |
| GLU_374@OE2 | THR_322@HG1 | THR_322@OG1 | 2.6782 | 164.9523 | 0.6160 |
| ARG_57@O | GLY_127@H | GLY_127@N | 2.8499 | 157.8217 | 0.6159 |
| ALA_200@O | ARG_215@H | ARG_215@N | 2.8475 | 158.8183 | 0.6138 |
| LEU_23@O | CYX_288@H | CYX_288@N | 2.8659 | 160.8749 | 0.6121 |
| TYR_137@O | ILE_169@H | ILE_169@N | 2.8506 | 158.8376 | 0.6113 |
| ILE_384@O | HID_395@H | HID_395@N | 2.8689 | 157.8386 | 0.6109 |
| GLU_273@O | LYS_280@H | LYS_280@N | 2.8588 | 161.1156 | 0.6082 |
| ASP_42@O | PHE_142@H | PHE_142@N | 2.8731 | 159.4562 | 0.6077 |
| ASN_47@O | GLU_138@H | GLU_138@N | 2.8567 | 158.4627 | 0.6028 |
| LYS_136@O | GLU_49@H | GLU_49@N | 2.8633 | 160.4193 | 0.6014 |
| ALA_140@O | LYS_44@H | LYS_44@N | 2.8764 | 161.7025 | 0.5967 |
| VAL_290@O | VAL_21@H | VAL_21@N | 2.8676 | 159.3197 | 0.5913 |
| VAL_91@O | ALA_117@H | ALA_117@N | 2.8643 | 157.7543 | 0.5856 |
| MET_48@O | LEU_281@H | LEU_281@N | 2.8565 | 159.3386 | 0.5845 |
| GLY_165@O | ILE_141@H | ILE_141@N | 2.8747 | 161.2356 | 0.5801 |
| THR_210@OG1 | THR_205@HG1 | THR_205@OG1 | 2.8162 | 163.1461 | 0.5792 |
| ILE_393@O | VAL_386@H | VAL_386@N | 2.8802 | 162.2580 | 0.5749 |
| VAL_279@O | ALA_50@H | ALA_50@N | 2.8738 | 160.2637 | 0.5717 |
| TYR_183@O | GLN_296@H | GLN_296@N | 2.8566 | 160.4253 | 0.5684 |
| ALA_117@O | VAL_91@H | VAL_91@N | 2.8711 | 159.9960 | 0.5678 |
| LEU_12@O | MET_34@H | MET_34@N | 2.8621 | 158.8899 | 0.5608 |
| LYS_337@O | CYX_305@H | CYX_305@N | 2.8692 | 160.7127 | 0.5527 |
| ARG_128@O | VAL_203@H | VAL_203@N | 2.8701 | 158.2069 | 0.5518 |
| THR_205@O | ILE_126@H | ILE_126@N | 2.8624 | 157.9194 | 0.5491 |
| VAL_386@O | ILE_393@H | ILE_393@N | 2.8734 | 161.0181 | 0.5471 |
| GLY_127@O | VAL_56@H | VAL_56@N | 2.8605 | 158.2764 | 0.5452 |
| ILE_126@O | THR_205@H | THR_205@N | 2.8744 | 159.9611 | 0.5417 |
| VAL_371@O | LEU_327@H | LEU_327@N | 2.8779 | 160.9417 | 0.5380 |
| ARG_215@O | MET_219@H | MET_219@N | 2.8656 | 158.7994 | 0.5250 |
| SER_342@O | VAL_352@H | VAL_352@N | 2.8674 | 160.3656 | 0.5201 |
| LEU_178@O | VAL_186@H | VAL_186@N | 2.8821 | 161.3523 | 0.5114 |
